# Malignancy Grade-Dependent Mapping of Metabolic Landscapes in Human Urothelial Bladder Cancer: Identification of Novel, Diagnostic, and Druggable Biomarkers

**DOI:** 10.3390/ijms21051892

**Published:** 2020-03-10

**Authors:** Aikaterini Iliou, Aristeidis Panagiotakis, Aikaterini F. Giannopoulou, Dimitra Benaki, Mariangela Kosmopoulou, Athanassios D. Velentzas, Ourania E. Tsitsilonis, Issidora S. Papassideri, Gerassimos E. Voutsinas, Eumorphia G. Konstantakou, Evagelos Gikas, Emmanuel Mikros, Dimitrios J. Stravopodis

**Affiliations:** 1 Section of Pharmaceutical Chemistry, Department of Pharmacy, School of Health Sciences, National and Kapodistrian University of Athens (NKUA), 15701 Athens, Greece; katerinail@pharm.uoa.gr (A.I.); arispan@pharm.uoa.gr (A.P.); dbenaki@pharm.uoa.gr (D.B.); mrgkosm@gmail.com (M.K.); 2 Section of Cell Biology and Biophysics, Department of Biology, School of Science, National and Kapodistrian University of Athens (NKUA), 15701 Athens, Greece; aigiann@biol.uoa.gr (A.F.G.); tveletz@biol.uoa.gr (A.D.V.); ipapasid@biol.uoa.gr (I.S.P.); 3 Section of Animal and Human Physiology, Department of Biology, School of Science, National and Kapodistrian University of Athens (NKUA), 15701 Athens, Greece; rtsitsil@biol.uoa.gr; 4 Laboratory of Molecular Carcinogenesis and Rare Disease Genetics, Institute of Biosciences and Applications, National Center for Scientific Research (NCSR) “Demokritos”, 15701 Athens, Greece; mvoutsin@bio.demokritos.gr; 5 Harvard Medical School, Massachusetts General Hospital Cancer Center (MGHCC), Charlestown, MA 021004, USA; ekonstantakou@mgh.harvard.edu

**Keywords:** biomarker, bladder, cancer, grade, metabolomics, MS, NMR

## Abstract

Background: Urothelial bladder cancer (UBC) is one of the cancers with the highest mortality rate and prevalence worldwide; however, the clinical management of the disease remains challenging. Metabolomics has emerged as a powerful tool with beneficial applications in cancer biology and thus can provide new insights on the underlying mechanisms of UBC progression and/or reveal novel diagnostic and therapeutic schemes. Methods: A collection of four human UBC cell lines that critically reflect the different malignancy grades of UBC was employed; RT4 (grade I), RT112 (grade II), T24 (grade III), and TCCSUP (grade IV). They were examined using Nuclear Magnetic Resonance, Mass Spectrometry, and advanced statistical approaches, with the goal of creating new metabolic profiles that are mechanistically associated with UBC progression toward metastasis. Results: Distinct metabolic profiles were observed for each cell line group, with T24 (grade III) cells exhibiting the most abundant metabolite contents. AMP and creatine phosphate were highly increased in the T24 cell line compared to the RT4 (grade I) cell line, indicating the major energetic transformation to which UBC cells are being subjected during metastasis. Thymosin *β*4 and *β*10 were also profiled with grade-specific patterns of expression, strongly suggesting the importance of actin-cytoskeleton dynamics for UBC advancement to metastatic and drug-tolerant forms. Conclusions: The present study unveils a novel and putatively druggable metabolic signature that holds strong promise for early diagnosis and the successful chemotherapy of UBC disease.

## 1. Introduction

Urothelial bladder cancer (UBC) exhibits the highest mortality rate worldwide, being categorized as the second most common genitourinary disease in the USA [1]. UBC still remains a major clinical challenge and its treatment mainly depends on early diagnosis [2]. It can be generally classified as a low-grade (I and II) non-muscle-invasive and a high-grade (III and IV) muscle-invasive disease that is prone to metastasis, based on histological differentiations from normal bladder cells [3]. One third of non-muscle-invasive UBCs progress to high(er) grades or stages of malignancy [4], which, along with the symptom-to-treatment delay for affected patients [5], compromise the therapeutic effectiveness and success of clinically applied regimens. UBC is characterized by high recurrence rates, with the continuous monitoring of patients being a medical practice of great importance [6]. Notably, despite their initial chemosensitivity, UBC patients will eventually develop chemoresistance due to tumors’ mutational heterogeneity, leading to a median survival expectancy of 13–19 months [7,8]. Thereby, novel, early diagnosis, and druggable biomarkers for UBC need to be promptly discovered.

Metabolomics, according to Professor J. Nicolson’s definition, is “the quantitative measurement of the dynamic and multi-parametric metabolic response of living systems to pathophysiological stimuli or genetic modifications” [9], with its great potential and promise in cancer research being continuously proved. The reprogramming of cellular metabolism seems to act as a strong force in tumorigenesis [10]. Malignant hallmarks, such as cell survival under stress conditions, as well as tumors’ ability to utilize nutrients and successfully encounter high-energy demands, are tightly correlated with metabolic alterations, thus indicating the major roles of metabolic landscapes in Cancer Biology [11,12,13,14]. Metabolic activities may significantly differ among distinct subtypes or malignancy grades/stages of the same type of cancer, leading to different metabolic networks and metabolomes [15,16]. Metabolomics has gained great value, power, and importance for cancer research, not only as a multifaceted tool in early diagnosis, but also as a valuable platform for the discovery of novel mechanisms controlling tumorigenesis, thus paving the way to new treatment strategies and therapies. Research on several cancers has significantly profited from the engagement of metabolomics technology. However, its application to early detection, progression, and the chemotherapeutic management of human malignancies and especially UBC remains still limited, and it needs to be further expanded [15,16,17,18,19,20].

Most metabolomics studies in UBC cell lines have focused on differentiating between normal bladder and UBC cells, and they have shown the importance of several metabolites involved in pathways related to energy production, such as fatty acids, amino acids, and organic acids. [21,22]. The effect of the oncometabolome on progression of the disease has recently emerged as a new field in UBC research [23,24,25,26]. Few studies used two cell lines, for low and high-grade UBC, in order to find distinct metabolic profiles between the two grades [27,28]. They observed that pyruvate consumption, as well as alanine and lactate levels, might be related to UBC aggressiveness, and they also suggested the role of fatty acid biosynthesis and amino acid metabolism in disease progression.

To further expand these studies, we herein engaged a collection of four human UBC cell lines that critically reflect the distinct de-differentiation stage, malignancy grade (I, II, III, and IV), mutational signature, genetic heterogeneity, metastatic capacity, and chemotherapeutic tolerance of UBC to thoroughly investigate the metabolic alterations to which UBC cells are being subjected during disease progression. Our findings are expected to shed light into mechanisms regulating the transition of normal to the oncogenic and, finally, metastatic cell phase. Employment of the two state-of-the-art analytical platforms, Nuclear Magnetic Resonance (NMR) and Mass Spectrometry (MS), which are complementary, allows the more comprehensive metabolic profiling of UBC, with the grade-specific metabolic signatures revealed essentially contributing to the significant advancement in the success, efficacy, and safety of conducted research. High-scale UBC cell culturing enabled the high-resolution metabolic landscaping, while the high density of cells upon harvesting portrayed tumor architectural organization in vivo. 

## 2. Results

In this study, a comparative NMR and LC-MS-mediated metabolic profiling was performed in four UBC human cell lines in order to thoroughly examine the effects of oncometabolome composition on progression of the disease.

### 2.1. NMR Analysis

**Figure 1:**
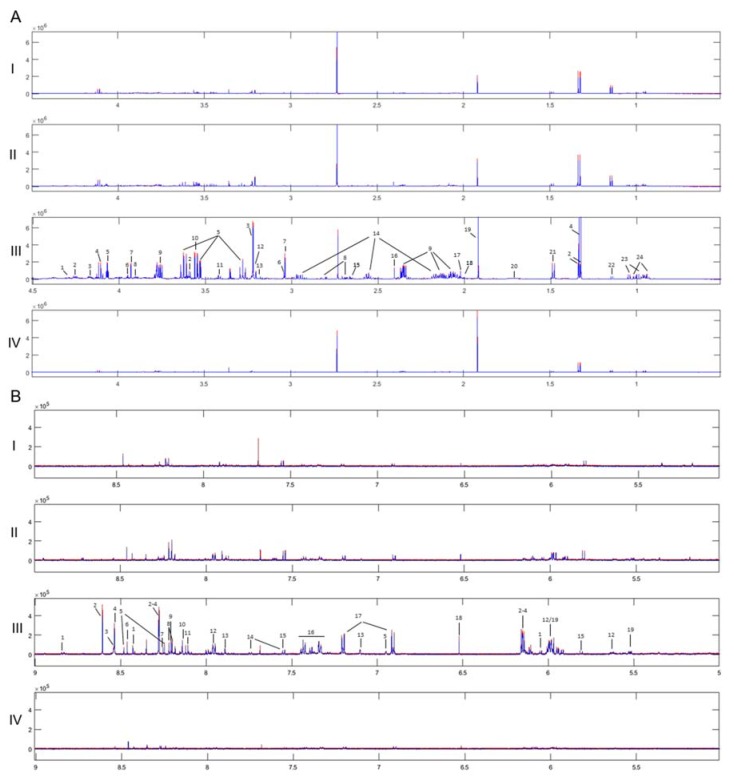
Average ^1^H NMR spectra of the four groups and their standard deviation, where very low within-group variability is observed. Annotation is shown on the most abundant spectrum of the grade III group. (I. Grade I, II. Grade II, III. Grade III, IV. Grade IV). Red Line: Average + standard deviation, Blue Line: Average–standard deviation. (**A**) Aliphatic region: 1. UDPs, 2. Threonine, 3. Choline phosphate, 4. Lactate, 5. Myo-inositol, 6. Creatine phosphate, 7. Creatine, 8. Aspartate, 9. Glutamate, 10. Glycine, 11. Taurine, 12. Choline, 13. β-Alanine, 14. Glutathione, 15. Malate, 16. Succinate, 17. N-Acetylglutamine, 18. Proline, 19. Acetate, 20. Leucine, 21. Alanine, 22. Propylene glycol, 23. Valine, 24. Isoleucine. (**B**) Aromatic region: 1. NAD+, 2. AMP, 3. ATP, 4. ADP, 5. NADH, 6. Formate, 7. Adenine, 8. Hypoxanthine, 9. Oxypurinol, 10. GTP, 11. UMP, 12. UDPs, 13. Histidine, 14. Tryptophan, 15. Uracil, 16. Phenylalanine, 17. Tyrosine, 18. Fumarate, 19. UDP-GlcNAc.

**Figure 2:**
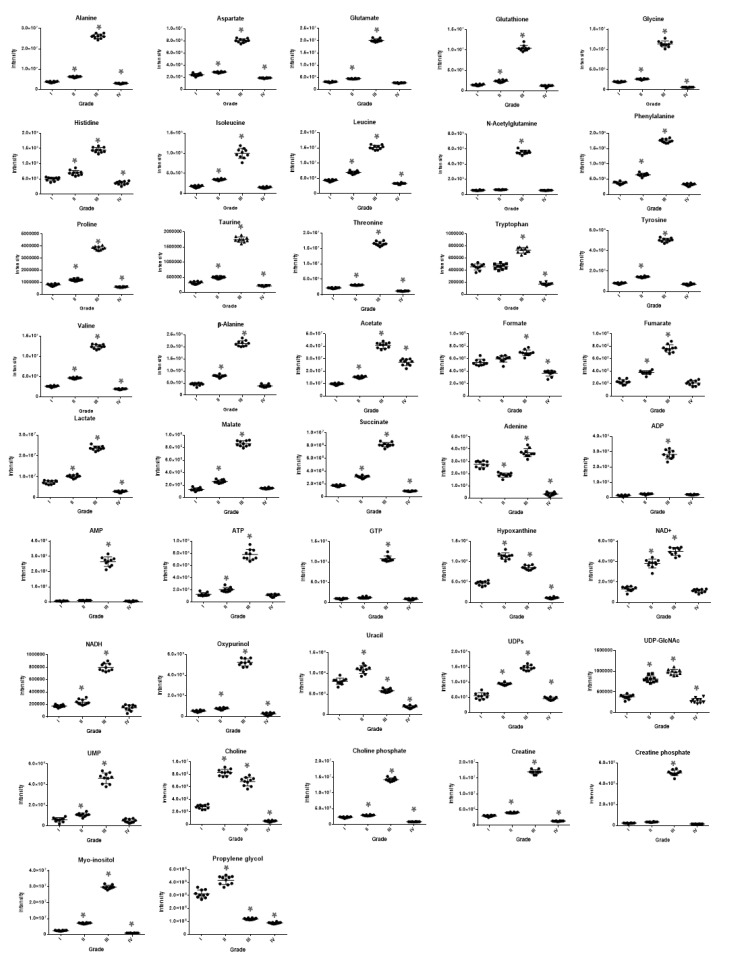
Boxplots of the 42 most significant metabolites, using ANOVA for the four-group urothelial bladder cancer (UBC) member comparisons. Myo-inositol, creatine phosphate, amino acids (e.g., histidine), organic acids (e.g., malate, succinate, and acetate), AXPs (AMP, ADP, and ATP), GTP, UMP, and oxypurinol follow the pattern of “metabolic inversion” that is being typified by their highly elevated contents in grade III (T24) group but reduced (restored) to the cell line-reference-like ones (RT4; grade I) in the grade IV (TCCSUP) UBC cell group. However, some metabolites are not subjected to the same pattern of grade-dependent deregulation (e.g., tryptophan, formate, uracil, adenine, propylene glycol, and choline). Remarkably, uracil and propylene glycol are presented with significant decreases during cellular transition from low to high grades (III and IV) of UBC malignancy.

The ^1^H 1D NMR spectra of the four UBC cell lines with the respective standard deviations obtained from each group are shown in Figure 1. The observed within-group variability has proved to be very low, thus confirming the precision and high-technical value of the procedure being followed for sample preparation and the subsequent instrumental analysis. In total, 42 metabolites have been identified (Supplementary Table S1), with the annotated and grade-dependent metabolites being described in Figure 2 as boxplot-type graphs.

**Figure 3:**
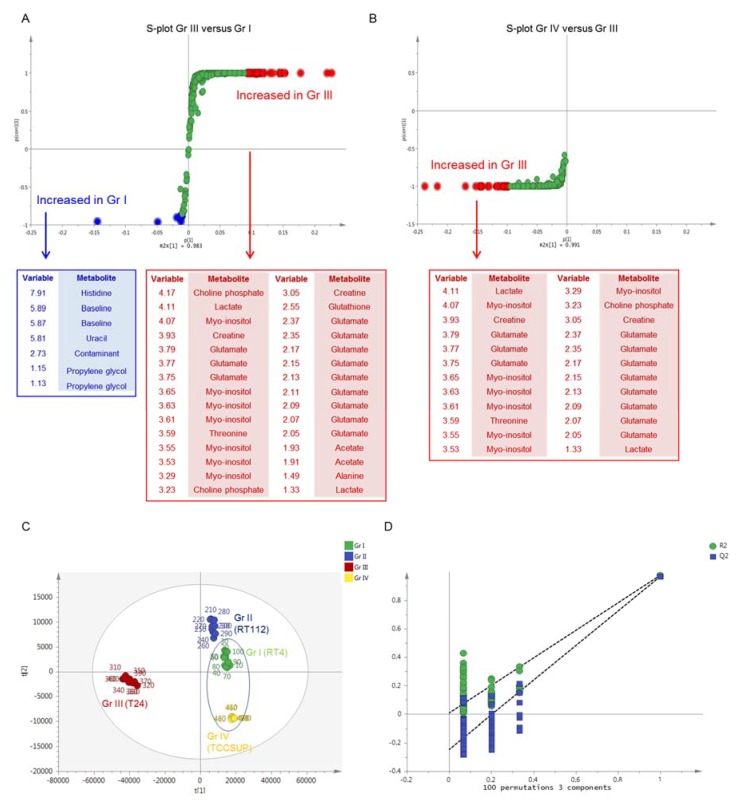
Multivariate analysis of ^1^H NMR spectra of the four UBC cell lines. (**A**) S-plot of Orthogonal Partial Least Square-Discriminant Analysis (OPLS-DA) model of grade I (RT4) versus grade III (T24) cell line and the respective loadings. Red dots: increased in grade III (T24), Blue dots: increased in grade I (RT4). (**B**) S-plot of OPLS-DA model of grade III (T24) versus grade IV (TCCSUP) cell line and the respective loadings. Red dots: increased in grade III (T24). (**C**) Scores plot of Partial Least Square-Discriminant Analysis (PLS-DA) model of the four UBC cell lines. Grade I (RT4) and grade IV (TCCSUP) groups seem unable to be discriminated, among each other, on the first principal component, while the grade III (T24) group is the most distant from the initial conditions on the same component. (**D**) Permutation test of the PLS-DA model of the four UBC cell lines. Gr: (malignancy) grade.

For the multivariate analysis, Principal Component Analysis (PCA) was first conducted to search for outliers and trends of discrimination. Νo outliers were detected (Hotteling eclipse, 95% confidence level), and a clear separation of the four UBC groups was observed (Supplementary Figure S1). Partial Least Square-Discriminant Analysis (PLS-DA) confirmed the clear separation of the examined groups, with good quality parameters and prediction power [R2X(cum) = 0.982 and Q2(cum) = 0.977]. Orthogonal Partial Least Square-Discriminant Analysis (OPLS-DA) pairwise comparisons were also performed to search for discriminatory variables for each grade-specific UBC cell line. The S-plot between grade I (RT4) and grade III (T24) UBC cells revealed that the T24 cell line exhibits notably upregulated levels of most metabolites, including, among others, amino acids (glutamate, alanine, threonine), organic acids (acetate, lactate), myo-inositol, creatine, and choline phosphate, while decreased contents of few metabolites (uracil, histidine, and propylene glycol) were observed (Figure 3A). The S-plot between grade III (T24) and grade IV (TCCSUP) UBC cells unveiled that the grade IV-specific levels of most metabolites were significantly reduced and restored almost to the grade I (RT4) respective levels, indicating a “metabolic inversion” effect during the advanced (late stage) UBC development (Figure 3B). This is observed in the PLS-DA scores plot of UBC groups, with RT4 and TCCSUP showing an inability to be discriminated on the first PC, while grade III (T24) was being proved as the most distant UBC group on the same component (Figure 3C). Validation of the PLS-DA model using permutation testing is shown in Figure 3D. Table 1 summarizes the results of univariate analysis of the identified metabolites, considering grade I (RT4) as the UBC cell line of reference (control) in order to explore malignancy grade-specific metabolic changes occurring during disease progression. Grade-dependent metabolites were detected and shown to be implicated in diverse metabolic pathways, such as amino acid metabolism, the tricarboxylic acid (TCA) cycle, and energy metabolism, as well as purine and pyrimidine metabolism. In accordance to the multivariate analysis, elevated contents of most metabolites are observed in grade III (T24), whereas they are restored (reduced) to their respective levels of the reference grade I group (RT4) [Fold Change (x) close to 1], or even lower, in the grade IV (TCCSUP) cells. Since the majority of metabolites (40 out of 42) exhibited significant increase in the grade III (T24) group, further investigation was performed in order to take into account dilution effects and metabolic dependencies. For the identified metabolites, all pairwise ratios were examined, and the fold change (x) of each metabolic trait was calculated. A heat map of the fold change (x) values of metabolic ratios between grade III (T24) and grade I (RT4) UBC cell groups is presented in Supplementary Figure S2. Among the 42 metabolites that were found to be significant, the pairwise ratios analysis highlighted the importance of a sub-collection containing 14 of them. Remarkably, uracil, hypoxanthine, adenine, tryptophan, propylene glycol, formate, UDPs, and choline ratios exhibited the highest decrease in grade III (T24) compared to grade I (RT4), whereas ADP, AMP, GTP, oxypurinol, creatine phosphate, and myo-inositol ratios showed the most significant increase in grade III (T24) [compared to grade I (RT4) reference group] cells. Importantly, the reduced metabolite contents, except for the uracil and propylene glycol ones, were found to be elevated in the analysis of metabolite concentrations only (expressed as signals). On the contrary, most of these metabolite ratios were shown to be decreased, which suggests that the analysis of metabolite concentrations only may lead to erroneous results when such enormous biological differences between compared groups are observed.

**Figure 4:**
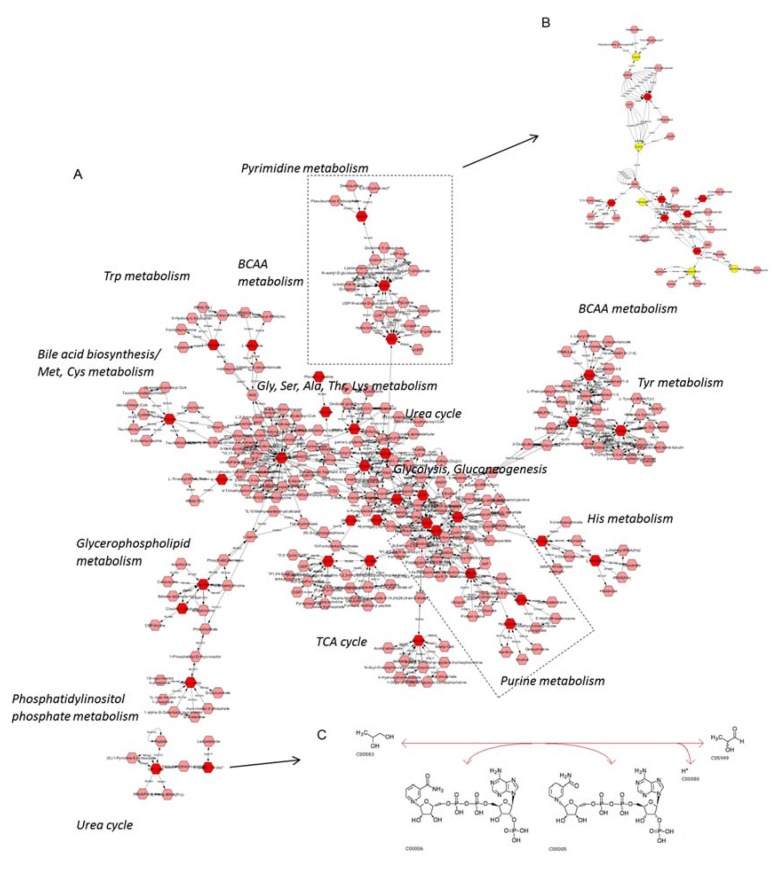
UBC metabolic network construction. (**A**) Hits (red) of the ^1^H NMR metabolomic analysis inserted into Cytoscape and the related pathways assembled. Purine/pyrimidine metabolism seems to play a crucial role in UBC progression. (**B**) Hits of the purine/pyrimidine metabolism in grade III (T24) versus grade I (RT4) cell group comparison. Red: increased in grade III (T24), Yellow: decreased in grade III (T24). (**C**) Propylene glycol (or propane-1, 2-diol) has emerged as a novel, putative biomarker, with the EC 1.1.1.21 enzyme reaction for propylene glycol having been taken from KEGG.

In order to systemically map and integrate the metabolic hits into their related biological pathways, the statistically significant metabolites were imported into CytoScape 3.7.0, using MetScape for metabolomics data visualization. Extracted pathway and metabolic course analyses are illustrated in Figure 4A. Among others, it seems that purine and pyrimidine metabolism (including the one of uracil) can critically control (positively or negatively) UBC progression to late-malignancy stages (Figure 4B). Importantly, propylene glycol (Kyoto Encyclopedia of Genes and Genomes (KEGG): 1, 2 propanediol), which is implicated in lactaldehyde metabolism, as shown in the constructed network (Figure 4A), may also be notably downregulated during UBC advancement toward metastasis (e.g., T24; grade III). Figure 4C describes the enzymatic reaction of aldo-keto reductase family 1 member B (AKR1B1), which is the enzyme that catalyzes the NADP-dependent conversion of propylene glycol to lactaldehyde in human (KEGG Reaction R02577, EC 1.1.1.21).

### 2.2. MS Analysis

Representative LC-MS chromatograms of each one of the four UBC cell lines are shown in Supplementary Figure S3. PCA scores plots, before and after filtering of the features based on the % Relative Standard Deviation in Quality Control samples (QCs) and QC-based Signal-correction Method (QC-RLSC) correction (Supplementary Figure S4A,B, respectively), for instrument drift and signal attenuation, remarkably lead to more consistent clustering of QCs after normalization, therefore improving the repeatability and accuracy of the study.

**Figure 5:**
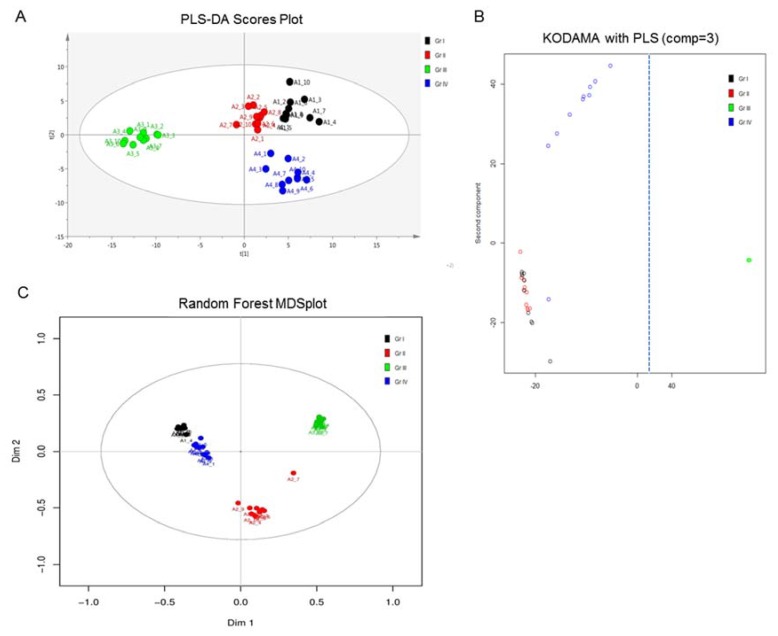
Comparison of the PLS-DA modeling of MS analysis, with KODAMA and Random Forest methods for the four UBC cell lines herein examined. All methods presented the greatest separation for the grade III (T24) group, highlighting T24 as the most suitable cell line to study the metabolic signature of highly malignant and strongly metastatic UBC disease. Black: grade I (RT4), Red: grade II (RT112), Green: grade III (T24), and Blue: grade IV (TCCSUP). (**A**) Principal Component (PC) scores plot from PLS-DA model. (**B**) PC scores plot from the KODAMA-PLS model. Figure is cut on the x-axis (dashed line), as grade III (T24) is far removed from the initial conditions. (**C**) Multidimensional Scaling (MDS) plot from the Random Forest model. Gr: (malignancy) grade.

In order to more deeply investigate for grade-specific variables among the four UBC cell groups that are able to uniquely reflect urothelial bladder-tumor pathologies (e.g., grade, stage, metastatic capacity, mutational load, and genetic heterogeneity), different multivariate approaches were employed, including the PLS-DA (SIMCA-P), KODAMA (R package), and Breiman’s Random-Forest (BR-F) (StatTarget) tools. The results obtained from multivariate analyses were evaluated based on their multi-ROC (Receiver Operating Characteristic) values (calculated for every MS feature) and are summarized in Table 2, wherein the features with an Area Under Curve (AUC) > 0.9 and their respective ranking after employing the different methodologies are shown. Loadings and Kruskal–Wallis ranking are the different platforms used by the Knowledge Discovery by Accuracy Maximization Analysis (KODAMA) algorithm for variable selection. The results described in Table 2 strongly suggest that KODAMA loadings and BF-R classification exhibit better performance for the four-group UBC-member comparisons than the classical PLS-DA model. Figure 5 presents the scores plot constructed using the three models. It is observed that the obtained results are in accordance with the ones derived from NMR analysis, with the grade III (T24) UBC cell group showing the highest separation on the first principal component in all three methods used. Detailed results for testing each variable’s importance using the different methodologies KODAMA, PLS-DA, and multi-ROC AUC are shown in Supplementary Table S2, while the first 50 ranked variables using BF-R are summarized in Supplementary Table S3.

Among the top ranked variables, peaks corresponding to two peptides have been herein identified, after deconvolution of multiple charged peaks and isotope clusters. Accordingly, for the first peptide, penta- (*m*/*z* 993.5027), exa- (*m*/*z* 828.0855), and epta- (*m*/*z* 709.9307) charged ions of *N*-Acetyl Thymosin *β*4 (T*β*4) were recognized, while the monoisotopic mass M + H = 4961.4792 was also obtained after deconvolution of the Electro-Spray Ionization (ESI) spectra. For the second peptide, Thymosin *β*10 (T*β*10), penta- (*m*/*z* 988.2166), exa- (*m*/*z* 823.5936), and epta- (*m*/*z* 705.7941) charged ions were identified, with the deconvoluted mass of 4934.5153 corresponding again to the *N*-Acetyl form of T*β*10 peptide. The previous identification of thymosin-type peptides [29] has revealed that the fragmentation of T*β*4 generates mainly fragments of the b series, while that of T*β*10 generates mainly fragments of the y series. The respective ions were searched in the MS/MS spectra of the penta- and exa-charged ions, using the peptide-fragmentation tool of mMass after noise filtering. The matched ions for T*β*4 and T*β*10 are described in Table 3.

**Figure 6:**
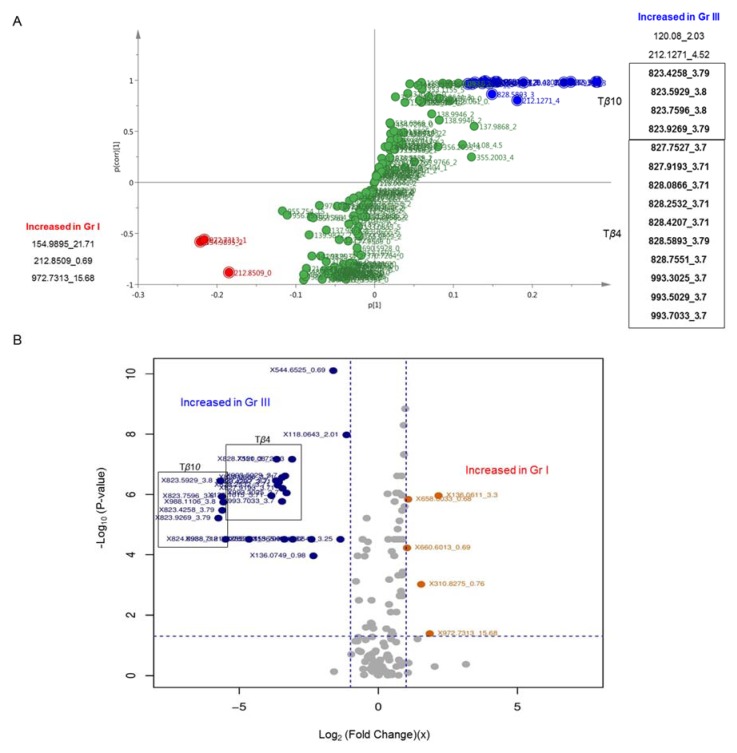
Comparison of the grade I (RT4) versus grade III (T24) cell groups and the significant MS analysis features. T*β*4 and T*β*10 charged peaks, and their respective isotopes, were found to be elevated in the grade III (T24) UBC cell group, using both univariate and multivariate approaches. Blue: increased in grade III (T24), Red: increased in grade I (RT4). (**A**) S-plot from OPLS-DA modeling multivariate approach. (**B**) Volcano plot of log_2_ [fold change (FC) (x)] versus *p*-values, and the selected variables with *p* < 0.05 and FC > 2, or < 0.5 (univariate approach). Gr: (malignancy) grade.

Pairwise comparisons, using OPLS-DA models and univariate analysis, have also been performed. Hence, T24 (grade III) were compared to RT4 (grade I) that serve as reference cells. Figure 6 presents the pairwise comparison between the lower (I) and higher (III) UBC grade, using both multivariate and univariate approaches. 

OPLS-DA analysis of these two UBC cell lines (RT4 and T24) was carried out to unveil important variables for their grade-specific and metabolic signature-dependent discrimination. In the OPLS-DA derived S-plot, features with the highest variation and reliability were selected. T*β*4 and T*β*10 were found to be strikingly elevated in the grade III (T24) UBC cell group. In the univariate analysis of the grade I (RT4) versus grade III (T24) UBC group, comparably similar results were obtained. T*β*10 peaks exhibit a log_2_ (FC) < −5, indicating a ca. 40-fold (x) increase in the grade III (T24) UBC cell group, while T*β*4 shows a log_2_ (FC) = −3, corresponding to a ca. 10-fold (x) increase for T24 cells, again.

## 3. Discussion

Cancer research has considerably benefited from cultured-cell metabolomics, which offers a number of advantages, such as minor ethical issues, cost-efficiency merit, and low biological variation, as compared to human biofluids [30,31]. In vitro metabolomics approaches facilitate the more integrated, systemic, and reliable experimental design, and, most importantly, the successful investigation of metabolic alterations that are directly and/or mechanistically linked to the tumorigenic process, and these may not be captured by blood and/or urine classical biochemical examination. However, there are still some challenges to cope with, such as the fast quenching of metabolism and data normalization [32,33]. To successfully encounter these challenges, we have chosen a grade-specific unique collection of UBC human cell lines that are able to largely maintain and thus mimic the pathology of the disease, including both its clinical features and molecular subtypes. Immediate freezing of UBC cultured cells ensured metabolism’s fast quenching equally for all the four cell lines examined, with RT4 (grade I) serving as the line of reference (control), due to its lowest malignancy grade among all. Data statistical processing, via the engagement of advanced algorithmic platforms, unearthed the major biological importance of our results to UBC progression toward tissue metastasis and refractory responses to chemotherapies.

To our knowledge, this is the first time all major UBC malignancy-grade cell types, I, II, III, and IV, with different mutational loads and metastatic proficiencies, are being metabolically fingerprinted, combining both NMR and LC-MS state-of-the-art technologies. Powerful and multifaceted statistical processing of the metabolic data ensures their biological validity and highlights the role of metabolomics pathways in UBC progression toward metastasis in vivo. Remarkably, each grade-specific UBC cell line has proved to carry its own unique metabolic signature that diagnostically, mechanistically, and therapeutically typifies each distinct tumorigenic phenotype examined. The question is whether the grade-dependent metabolome governs UBC pathology and aggressiveness, or, vice versa, the advancement to metastatic state(s) compels the acquisition of metabolic aberrations and the generation of derailed UBC metabolomes. It must be the grade-specific molecular signature of each UBC cell type that defines its unique metabolic landscape. However, the four cell lines herein examined carry different mutational profiles, with some mutations being likely unrelated (due to their generation stochasticity) to UBC aggressiveness. Hence, the possibility that these (non-causative for tumor features) mutations could be linked to some of the observed metabolic alterations cannot be excluded. Nevertheless, the major mutation contents of the cell lines used are indeed grade dependent, and they can be classified according to the UBC grade, metastatic capacity, and general disease pathology. In any case, the high grade-specific oncometabolomes (e.g., of T24) seem to contain a number of critical metabolites that can serve as novel and powerful biomarkers for both the diagnosis and drug-mediated clinical management of UBC disease. 

In this study, a four-cell-line group of a human UBC model has been in vitro engaged to thoroughly investigate the metabolic alterations that tumor cells likely undergo during UBC advancement, thus highlighting the importance of a malignancy grade, mutational signature, de-differentiation state and metastatic activity to “diagnostic biomarkering” and “targeted therapeutic drugging” of the disease. The four human UBC cell lines that have been analyzed critically reflect the distinct de-differentiation state and malignancy grade (I–IV) of UBC in vivo. Our results clearly indicate that T24 (grade III) can serve as a powerful, informative, and versatile cell-line system that can be successfully exploited to illuminate the role of metabolic reprogramming in UBC progression toward metastasis. It seems that the elevated contents of most metabolites specifically identified in T24 are tightly associated with the highly oncogenic character of the cells. Notably, PCA analysis showed that the grade III-specific T24-cell group exhibits the clearest separation on the first principal component, both in NMR and MS analysis, thus explaining the maximal amount of variation for its discrimination among the four cell groups. These results are strongly supported by biological data interpretations, since T24 cells bear a heavy mutational load and strong tumorigenic capacity. They are characterized by the mutant, oncogenic, form H-RAS^G12V^ and the disruption of stress-induced activation of mutant p53 (ΔΥ^126^) protein [34]. Hence, the aberrant signaling activity of H-RAS^G12V^, in a cellular environment that lacks the genome-protecting properties of p53, may render T24 cells susceptible to severe metabolic reprogramming, with grade III-specific metabolites fostering and promoting highly tumorigenic features and pathologies, including genetic instability, clonal heterogeneity, chemoresistance, immune escape, and organ metastasis (tissue invasion). Interestingly, a role of *H-RAS^G12V^* oncogene (*RAS*) in metabolic reprogramming during early mammary carcinogenesis has been previously reported, with the MCF10A-*RAS* transfected human breast epithelial cells exhibiting enhanced glycolytic activity and lactate production [35], consistently with the cancer hallmark of the classic “Warburg effect” [36,37]. Comparative genomics evidence that indicates that the T24 cell line (https://depmap.org/portal/cell_line/ACH-000018?tab=mutation) reliably reflects the mutational profile of muscle-invasive bladder cancer patients (https://www.cbioportal.org; Bladder Cancer, TCGA Cell 2017, 413 Total Samples) supports and increases the utilization of T24 as a valid, pre-clinical, cell-model system for advanced bladder cancer research in diagnosis and therapy. Remarkably, besides the *H-RAS* and *TP53* (encodes p53) mutated oncogenes, T24 cells share with muscle-invasive UBC patients multiple genes carrying molecular alterations, including (among others) the *KDM6A*, *MAGEF1*, *DIDO1*, and *EP300* ones, with 37%, 31%, 30% and 30% of detection frequency, respectively, in the UBC patient cohort studied (Supplementary Table S4). 

Among all UBC–cell pairs, embracing different grades being compared [e.g., II versus I, III versus I, and IV versus I; I (RT4) herein serves as the cell line of reference], only the grade III (T24) versus grade I (RT4) proved to significantly differ in the majority of metabolite contents examined, thus indicating the major role of the T24-specific mutational signature (including H-RAS^G12V^ and p53^ΔY126^) in metabolome composition and its oncogenic proficiency. The surprising resemblance between grade I (RT4) and grade IV (TCCSUP) metabolic profiles strongly suggests the engagement of a “metabolic inversion” process that likely favors late-metastasis cells to successfully encounter energetic challenges, nutritional demands, and oxygen deprivations. It is possible that a pre-metastatic UBC cell, after it becomes metastatic and invades other (proximal or/and distal) tissues/organs, will undergo a dramatic metabolic reprogramming to suppress its ability for a second metastasis event. If so, it may always be the primary tumor mass that feeds metastasis, with a metastatic-cell clonal population likely colonizing tissues outside the urinary bladder only once. In accordance, cell dissemination seems to occur during the early stages of tumor evolution, with cells from early and low-density lesions displaying more “stemness” features, migrating more and founding more metastases than cells derived from dense and advanced tumors [38,39,40].

The majority of previous UBC metabolomics reports have underlined the importance of amino acids to urothelium oncometabolome, with most amino acid levels being upregulated compared to controls; similar differential patterns were described for advanced UBC stage(s) compared to early stage(s). Interestingly, increased contents of glutathione were found in UBC cell lines, while glycine was elevated in the tumorigenic cells as well [21,22]. Alanine was also increased in the TCCSUP (high-grade) cell line compared to the RT4 (low grade) one [28]. An abundance of amino acids is important for the proliferative cancer cells, not only as substrates of protein synthesis, but also for energy generation, cellular redox homeostasis, and nucleotide biosynthesis [41]. Pyruvate consumption and alterations in glycolytic profile have also been related to UBC aggressiveness, as anaerobic conditions in high(er) grade UBC favor the conversion of pyruvate to lactate, or alanine [28]. In our study, and in accordance with previous results, T24 (grade III) exhibited elevated contents of totally 17 amino acids and derivatives, with the highest increase being detected for N-acetylglutamine, glutathione, alanine, and glutamate, together with a 3x increase of lactate. Nucleotides are also involved in biomass build-up observed in cancer cells and in the changes of ATP concentrations that indicate the energetic status (usually crisis) of tumors [42]. In serum samples of UBC patients, the raised levels of hypoxanthine and reduced levels of uracil indicated a perturbed breakdown of purine nucleotides, favoring the purine synthesis pathway (reviewed in ref. [17]). Notably, herein, purine metabolism has emerged as one of the most important networks in our *in silico* analysis (Figure 4), thus highlighting its major contribution to UBC aggressiveness and progression toward metastasis. Increased contents of hypoxanthine and adenosine nucleotides (AMP/ADP/ATP), with up to a 34x striking increase for AMP, were observed in T24 (grade III), but they were surprisingly restored (again) to the initial (RT4-like) levels in TCCSUP (grade IV) cells. In a study of urine samples derived from UBC patients, it has been suggested that the high(er) levels of choline phosphate may reflect increased lipid membrane remodeling, which has also been reported for other cancers [24,42]. Choline phosphate and, to lower extent, choline contents were also increased in our T24 (grade III) cell-line group, with a 6x and 2x rise, respectively. As expected, the elevated contents of these metabolites were reduced in TCCSUP (grade IV) cells, indicating the activation of a “metabolic inversion” effect. Regarding the two major metabolites involved in osmoregulation and volume regulation, taurine and myo-inositol, contradictory results were previously reported, with either upregulated or downregulated levels detected (reviewed in ref. [17,42]), likely implicating the role of cellular micro-environments in metabolome compositions. Interestingly, herein, significantly elevated contents of myo-inositol (12x) and taurine (5x) were observed in T24 (grade III), indicating the high grade-dependent ability of UBC to regulate osmosis and volume, in favor of promoting cell motility and tumor metastasis.

Taking into account that the majority of the identified metabolites (40 out of 42) were increased in the grade III (T24) group, multivariate analysis and the use of pairwise ratios were employed in order to highlight metabolites with major alterations. Notably, multivariate analysis of the obtained NMR data indicated the importance of, among others, glutamate and myo-inositol level deregulations to UBC pathology, while AXPs (AMP, ADP and ATP), GTP, oxypurinol, and creatine phosphate content increases were presented with the strongest statistical significance in the grade III/I cell pair (III/I). The use of pairwise ratios allowed us to account for dilution and signal instability effects and to unveil alterations that are masked by the “metabolic inversion” phenomenon. Increased ratios were found in III/I for metabolites that were also profiled with a “metabolic inversion” effect in IV/I (e.g., AMP, oxypurinol, myo-inositol, and creatine phosphate); thus, they might critically contribute to UBC advancement. On the other hand, decreased ratios were observed in III/I for several metabolites that were also presented with downregulated proportions in IV/I (e.g., uracil and propylene glycol) and, as such, they could presumably function as UBC-specific “oncosuppressing” modulators/inducers. Further discussion will focus on those metabolites that were herein identified as the most important by both (NMR and LC-MS) applied technologies, and especially the ones with the highest fold change (x) in metabolite levels and the highest alterations in all pairwise metabolic ratios.

Regarding the top two metabolites with the highest elevation contents in grade III (T24) versus grade I (RT4) UBC cells, creatine phosphate and AMP, they were remarkably presented with 23x and 34x positive change (upregulation), respectively (Table 1). Strikingly, by taking uracil as the metabolite of reference (since it was downregulated both in III/I and IV/I cell pairs), their respective fold changes (x) were further increased up to 32x and 50x values (Supplementary Table S5). Since colon cancer-derived liver metastases carry higher creatine kinase brain type (CKB) levels compared to primary tumors [43,44], UBC metastatic populations, in order to overcome hypoxia and other metabolic stresses, may also upregulate CKB expression or/and activity. This allows (after CKB secretion) energy to be likely captured from the extracellular ATP-mediated generation of creatine phosphate and its subsequent SLC6A8-dependent import into metastatic cells to regenerate ATP. Similarly to colon cancer cells [45], hypoxic UBC cells, in the absence of functional Hypoxia-Inducible Factor 1-alpha (HIF1α) (a key regulator of hypoxia response) pathway, could adapt their energy metabolism via the upregulation of creatine metabolism (and synthesis), thus opening a new chemotherapeutic window for metastatic UBC targeting and management in the clinic. Most importantly, T24 (grade III) cells contain a mutant version of the EP300 (KAT3B/p300) transcriptional co-activator (Supplementary Table S4) (https://depmap.org/portal/cell_line/ACH-000018?tab=mutation), which, in its wild-type form, is required for the transcriptional activation of HIF1α-target genes [46]. This indicates their competence to survive and grow in adverse hypoxic environments through the engagement of HIF1α-independent, but likely creatine-dependent, metabolic pathways. Given that the calculated fold change (x) in the III/I pair for ADP is 20.76x, which is a value close to the 22.99x of creatine phosphate (Table 1) (an approximately 1:1 molecular stoichiometry), T24 (grade III) cells may utilize intracellular creatine phosphate as a phosphate donor to the available ADP to finally produce ATP.

The remarkably elevated content of AMP in T24 (grade III) versus RT4 (grade I) cells indicates its major value for UBC progression toward chemoresistance and metastasis. High levels of ADP and AMP in a cell undergoing energetic stress/crisis cause significant increase in the AMPK kinase activity [47]. Activated AMPK phosphorylates a number of target substrates to regulate cell growth, metabolism, and autophagy [48]. Interestingly, activated H-RAS (G12V) requires autophagy for the maintenance of tumorigenesis [49]. Therefore, it seems that the major AMP/ADP/ATP (AXPs) metabolic reprogramming specifically observed in grade III (T24) cells may be tightly related to the aberrant signaling of the H-RAS^G12V^ mutant oncoprotein. If so, H-RAS^G12V^–AMP/ADP–AMPK–autophagy must operate as an indispensable axis for UBC cell survival and growth in unfavorable and adverse (e.g., hypoxic or nutritionally deprived) environments. Since we have previously shown that T24 cells are characterized by constitutively activated basal autophagy [34], the H-RAS^G12V^-induced intracellular energetic stress, in the form of AMP (and ADP) highly upregulated levels (this study), serves as a valuable and powerful metabolic biomarker, with its implicated enzymes/regulators likely opening a new therapeutic window for UBC metastasis and drug tolerance.

Intriguingly, propylene glycol and uracil herein emerged as metabolites that were downregulated in both III/I and IV/I grade cell pairs. Especially for propylene glycol, a prominent reduction in its intracellular contents was observed for both T24 (grade III) and TCCSUP (grade IV) compared to RT4 (grade I) cells of reference. Propylene glycol is produced by the conversion of pyruvaldehyde to lactaldehyde, which is then converted to propylene glycol via the aldehyde reductase mediation. Aldo-keto reductases family 1 members A1 and B1 (AKR1A1 and AKR1B1) are part of the Aldo-keto reductase superfamily and catalyze the reduction of several aldehydes. Data derived from the TCGA (The Cancer Genome Atlas) platform (https://www.cbioportal.org) strengthen our interpretation for perturbed (compromised) aldehyde reductase activities in advanced metastatic UBC disease, with 16% and 7% of the examined muscle-invasive UBC patients (Bladder Cancer, Cell 2017, z: 1.5) exhibiting deregulated expression/activity of the AKR1A1 and AKR1B1 enzymes, respectively, and 2.67% of them bearing low mRNA levels of the *AKR1A1* gene (https://www.cbioportal.org). Thereby, it seems that T24 may have originated from a patient with a molecular signature of downregulated *AKR1A1* gene expression in the tumor cells.

A major advantage (and novelty) of the present study is the employment of two complementary, state-of-the-art, analytical platforms: Nuclear Magnetic Resonance (NMR) and Mass Spectrometry (MS). Their successful combination offers a comprehensive metabolic characterization that covers a variety of chemical structures and concentrations of profiled metabolites. Strikingly, application of the MS-based metabolomics technology led to the detection and identification of novel molecules that could significantly contribute to UBC progression toward metastasis. Two peptides, T*β*4 and T*β*10, were identified with a high confidence level, and proved to be statistically significant both in the discrimination of the four (I, II, III, and IV) groups and of the low(er) or high(er) malignancy grade pairwise comparisons. Members of the *β* thymosin family form a (1:1) complex with the monomeric G-Actin protein, acting as its sequestration peptides, thus critically controlling actin cytoskeleton dynamics [50]. Furthermore, in contrast to T*β*4, T*β*10 can also directly bind to RAS, inhibiting its signaling activity, in an endothelial cell environment [51]. Nevertheless, in a T24-specific cellular setting, T*β*10 could no longer interact with the mutant H-RAS^G12V^ form, thus presumably releasing its aberrant signaling function(s) to drive the oncogenic and metastatic phenotypes of grade III UBC cells. Hence, the strikingly elevated contents of T*β*4 (10x) and T*β*10 (40x) in T24 (grade III) compared to RT4 (grade I; reference) cell line strongly suggest their actin cytoskeleton remodeling-dependent role in UBC advancement to metastasis, with oncogenic H-RAS^G12V^ (after its presumable release from T*β*10) also orchestrating UBC aggressiveness and drug/radiation resistance. Accordingly, T*β*10 overexpression correlates with the poor prognosis and progression of UBC disease [52], while T*β*4 expression is associated with clinical outcomes and clinicopathological parameters of UBC patients (it is significantly increased in UBC patients versus normal (control) volunteers) [53]. Most importantly, TCGA-derived data unveiled 6% of muscle-invasive UBC cases (bladder cancer, “amplification” and “mRNA high”, Cell 2017, z: 1.5) to be carrying upregulated levels of *TMSB10* gene (encodes T*β*10) expression (https://www.cbioportal.org), thus indicating the in vivo importance of T*β*10 (and T*β*4) to UBC progression toward high-malignancy grades and aggressive metastatic stages that are characterized by resistance to (chemo/radio)therapy, shorter survival of patients, and, generally, poor prognosis.

## 4. Materials and Methods 

### 4.1. Chemicals and Reagents

The detailed information for chemicals and reagents is listed in Supplementary Materials.

### 4.2. Cell Lines and Culture Conditions

The UBC human cell lines RT4, RT112, T24, and TCCSUP were used in the present study. RT4 was obtained from ECACC-Sigma-Aldrich (Munich, Germany). RT112 was kindly provided by Professor J.R. Masters (London, England, UK). T24 and TCCSUP were purchased from ATCC-LGC Standards GmbH (Wesel, Germany). All cell lines were derived from urothelial cell carcinomas of human urinary bladder, with RT4 being classified as malignancy grade I, RT112 as grade I–II (II), T24 as grade III, and TCCSUP as grade IV. All four UBC cell lines were authenticated mainly using in-house established technologies. Cell authentication was based on several combinational criteria, such as the cell-specific morphology, growth rate, nutritional requirement, mitotic index, immunofluorescence pattern (e.g., Epithelial–Mesenchymal Transition (EMT) phenotype), mutational load (e.g., *H-RAS* and *TP53*), signaling activity (e.g., Akt and p44/42 MAPK {ERK1/2}), protein content, gene-expression profiling, drug response, motility (e.g., wound-healing assay) and tumorigenicity (e.g., xenograft in Severe Combined ImmunoDeficient (SCID) mouse). All four UBC human cell lines were being tested periodically, but, most importantly, they were thoroughly examined just before the commencement of their large-scale growth for the herein implemented metabolomics analysis. The detailed culture conditions are described in the Supplementary Materials.

### 4.3. Cell Collection and Storage

The analytical protocols of cell collection and storage are described in the Supplementary Materials.

### 4.4. Metabolomics Experiments

The detailed protocols of metabolomics experiments (e.g., metabolite extraction, sample preparation, NMR, and MS analysis) are described in the Supplementary Materials.

### 4.5. Data Preprocessing

The binning of NMR spectra was conducted (0.001/0.02 ppm) using the AMIX software. Regions of water and contaminations being observed at the blank solutions were removed from the analysis. Pairwise ratios of the metabolites identified in NMR analysis were also calculated (42 × 42 = 1.764 metabolic traits). In MS analysis, data preprocessing was performed using the XCMS online. Peak-based normalization was applied in order to correct data within the batch experiment. Specifically, a QC-based signal-correction method (QC-RLSC), engaging the non-linear local polynomial regression (LOESS), was performed using the MetaX R package [54]. KNN (*K*-Nearest Neighbor) imputation for missing values and filtering of features based on the % Relative Standard Deviation (RSD) in QCs were applied.

### 4.6. Metabolite/Pathway Identification

The Chenomx NMR suite (Chenomx Inc., Alberta, Canada) was utilized for the NMR-mediated metabolite identification. For the recognition of peptides, the mMass peptide tool v. 5.5.0 was suitably employed [55]. The Cytoscape platform was engaged for visualizing molecular networks of significant metabolites derived from the NMR analysis [56,57].

### 4.7. Statistical Analysis

Principal Component Analysis (PCA), Partial Least Square-Discriminant Analysis (PLS-DA), and Orthogonal Partial Least Square-Discriminant Analysis (OPLS-DA), using SIMCA-P 14.0 (Umetrics, Umea, Sweden), were suitably applied. The quality of obtained models was assessed via R2X (variance explained by X Matrix) and Q2 (Goodness of prediction) obtained by 7x cross-validation, parameters, and permutation test results (100 random permutations for the PLS-DA and OPLS-DA models). The Knowledge Discovery by Accuracy Maximization Analysis (KODAMA) R package [58] was performed for the unsupervised extraction of variables in the MS analysis. The selected classifier was PLS-DA, and the procedure was repeated 100 times. Two methods implemented in the same package were used for the ranking of variables’ importance; Kruskal–Wallis test and the model’s loadings. Breiman’s Random-Forest (BR-F) algorithm was also evaluated using the StatTarget tool [59]. A number of 500 grown trees and 20 permutations were imported as model parameters. Multi-ROC AUC was used for the evaluation of methods performance using the multi-ROC R package [60].

## 5. Conclusions

In the present study, the metabolic landscapes of grade I, II, III, and IV UBC human cell lines were extensively mapped. Obtained results indicated a prominently perturbed amino acid and purine/pyrimidine metabolism with a remarkable increase of most metabolites being identified in grade III (T24) UBC cells, using RT4 (grade I) as the line of reference. Surprisingly, insignificant changes were observed for grade IV (TCCSUP) cells, thus implying the activation of a “metabolic inversion” process. T24 (grade III) has proved the most powerful and versatile cell line, which is able to accurately and reliably unveil the metabolic signature of highly malignant and strongly metastatic UBC pathology. “Metabolic inversion” has to be mechanistically investigated, in order to open new chemotherapeutic windows for UBC advancement to drug-resistant metastasis. Analysis of NMR-derived metabolite contents and ratios showed significant perturbations in purine and pyrimidine metabolism, while MS analysis demonstrated the importance of T*β*4 and T*β*10 peptides to UBC progression toward metastasis. AMP (and ADP) highly upregulated levels indicate the H-RAS^G12V^-induced intracellular energetic stress/crisis, and they can serve as valuable metabolic biomarkers, while the remarkably elevated contents of T*β*4 and T*β*10 in the T24 (grade III) compared to RT4 (grade I) cell line strongly suggest their actin cytoskeleton remodeling-dependent role in UBC advancement to metastasis and drug tolerance. Furthermore, decreased levels of propylene glycol are indicative of dysregulated *AKR1A1* gene expression in the tumor cells of urinary bladder, thus rendering it (propylene glycol) as a potentially significant biomarker.

Our work also made use of novel statistical approaches for metabolomics data analysis. Metabolic ratios are strongly suggested to account for the size effect present in the data and to highlight novel metabolic pathways. Hence, their combined employment and biological interpretation are crucial in order to avoid erroneous results. A common bottleneck in the untargeted MS analysis is the selection of important variables. We herein compared the typical PLS-DA method used in metabolomics analyses with other statistical tools carrying different merits and drawbacks (e.g., KODAMA and BF-R). Our results underline the importance of either pairwise comparisons or the implementation of more sophisticated multivariate approaches, such as the Random Forest models, which may exhibit better performances.

Most importantly, the integration of high-resolution metabolic maps with high-scale proteomic profiles containing enzymes/regulators that are able to control the homeostasis of grade-dependent UBC intracellular metabolites will shed light on mechanisms of urothelial bladder tumorigenesis, and they will benefit our tools in terms of the safe, efficient, and generally successful (including reduced medical costs) diagnostic and therapeutic management of the advanced UBC disease in the clinic.

## Figures and Tables

**Table 1:** The identified metabolites and the calculated fold change (×) during UBC progression, taking grade I (RT4) as the cell group of reference. A three-color gradient is applied, depending on the fold change (×) value. Red denotes the highest value(s). Gr: (malignancy) grade.

Metabolites	Gr II/Gr I	Gr III/Gr I	Gr IV/Gr I
Alanine	1.66	6.99	0.81
Aspartate	1.18	3.33	0.78
Glutamate	1.41	6.44	0.88
Glutathione	1.65	7.18	0.87
Glycine	1.33	5.71	0.32
Histidine	1.41	2.91	0.74
Isoleucine	1.96	5.62	0.86
Leucine	1.61	3.51	0.77
N-Acetylglutamine	1.14	10.17	0.99
Phenylalanine	1.82	5.92	0.86
Proline	1.5	4.77	0.75
Taurine	1.55	5.5	0.67
Threonine	1.42	5.38	0.74
Tryptophan	1.01	1.6	0.37
Tyrosine	1.77	6.25	0.88
Valine	1.79	4.81	0.74
β-Alanine	1.68	4.54	0.82
Acetate	1.56	4.17	2.78
Formate	1.13	1.28	0.68
Fumarate	1.65	3.34	0.92
Lactate	1.39	3.22	0.39
Malate	1.95	6.5	1.12
Succinate	1.79	4.65	0.51
Adenine	0.68	1.33	0.12
ADP	1.76	20.76	1.49
AMP	1.45	34.33	0.79
ATP	1.6	6.1	0.88
GTP	1.29	11.31	0.88
Hypoxanthine	2.41	1.81	0.24
NAD+	2.86	3.76	0.86
NADH	1.36	4.62	0.83
Oxypurinol	1.46	10.16	0.45
Uracil	1.36	0.72	0.23
UDPs	1.71	2.66	0.83
UDP-GlcNAc	2.43	3.25	0.75
UMP	1.82	7.77	0.82
Choline	2.96	2.44	0.19
Choline phosphate	1.27	6.26	0.37
Creatine	1.35	5.73	0.45
Creatine phosphate	1.51	22.99	0.66
Myo-inositol	2.89	12.06	0.37
Propylene glycol	1.34	0.38	0.29

**Table 2:** MS features with multi-ROC AUC > 0.9 and their ranking, based on different multivariate approaches. Knowledge Discovery by Accuracy Maximization Analysis (KODAMA) loadings and Breiman’s Random-Forest (BF-R) classification exhibit better performance for the four-group UBC-member comparisons than the classical PLS-DA model.

Feature	Multi-ROC AUC	Variable Importance in Projection (VIP) (PLS-DA)	Loadings Ranking	Kruskal Ranking	Random Forest (RF) (*p*-Value)
993.7033_3.7	0.995	56	11	54	2
828.2532_3.71	0.982	77	6	58	5
129.1015_3.71	0.98	79	4	61	2
993.3025_3.7	0.973	66	5	53	6
348.7831_0.69	0.972	22	3	1	7
993.5029_3.7	0.972	58	13	66	11
828.0866_3.71	0.96	69	9	57	20
136.0611_3.3	0.955	1	66	11	8
827.9193_3.71	0.952	71	14	65	12
828.4207_3.71	0.95	74	7	33	9
696.5591_0.69	0.942	49	52	17	41
823.9269_3.79	0.938	55	12	24	16
824.0938_3.8	0.938	52	32	70	13
120.08_2.03	0.935	99	57	4	15
823.4258_3.79	0.932	51	2	3	16
823.5929_3.8	0.932	41	15	31	5
823.7596_3.8	0.928	53	23	25	18
380.7729_0.69	0.925	86	49	28	38
118.0643_2.01	0.922	39	37	16	28
988.1106_3.8	0.917	54	20	12	1
828.7551_3.7	0.913	67	10	69	18
622.646_0.68	0.912	65	16	8	34
350.7812_0.69	0.91	47	26	20	31
212.8509_0.69	0.908	19	38	36	30
828.5893_3.79	0.908	112	30	55	14
988.7121_3.78	0.907	62	17	47	26
330.7728_0.66	0.902	36	33	13	45
486.713_0.66	0.902	34	28	6	>50
827.7527_3.7	0.9	84	21	79	22

**Table 3:** Matched ions of b series, with the MS/MS fragmentation profile of the penta- (*m*/*z* 993.5027) and exa- (*m*/*z* 828.0855) charged (acetylated) thymosin *β*4 (T*β*4), and matched ions of the y series, with the MS/MS fragmentation profile of the penta- (*m*/*z* 988.2166) and exa- (*m*/*z* 823.5936) charged (acetylated) thymosin *β*10 (T*β*10).

**Ions of b Series, (Acetylated) Thymosin *β*4 (T*β*4)**					
***m*/*z***	**Ion**	**z**	**Sequence**	**Error (Da)**	**Deconvoluted Mass**
774.9006	b13	2	.SDKPDMAEIEKFD.k [1xAcetyl]	0.053	1547.696
946.616	b16	2	.SDKPDMAEIEKFDKSK.l [1xAcetyl]	0.157	1890.9178
1067.2304	b18	2	.SDKPDMAEIEKFDKSKLK.k [1xAcetyl]	0.182	2132.097
831.2934	b21	3	.SDKPDMAEIEKFDKSKLKKTE.t [1xAcetyl]	0.197	2490.2895
355.2822	b27	9	.SDKPDMAEIEKFDKSKLKKTETQEKNP.l [1xAcetyl]	0.096	3187.6722
850.3742	b29	4	.SDKPDMAEIEKFDKSKLKKTETQEKNPLP.s [1xAcetyl]	−0.069	3397.7732
936.3339	b32	4	.SDKPDMAEIEKFDKSKLKKTETQEKNPLPSKE.t [1xAcetyl]	−0.152	3741.9428
869.5772	b37	5	.SDKPDMAEIEKFDKSKLKKTETQEKNPLPSKETIEQE.k [1xAcetyl]	0.132	4342.2255
1086.6825	b37	4	.SDKPDMAEIEKFDKSKLKKTETQEKNPLPSKETIEQE.k [1xAcetyl]	0.128	4342.218
1164.2402	b40-NH_3_	4	.SDKPDMAEIEKFDKSKLKKTETQEKNPLPSKETIEQEKQA.g [1xAcetyl]	0.145	4669.4134
788.6117	b41	6	.SDKPDMAEIEKFDKSKLKKTETQEKNPLPSKETIEQEKQAG.e [1xAcetyl]	−0.129	4726.4448
1182.801	b41	4	.SDKPDMAEIEKFDKSKLKKTETQEKNPLPSKETIEQEKQAG.e [1xAcetyl]	0.194	4726.4304
971.9887	b42	5	.SDKPDMAEIEKFDKSKLKKTETQEKNPLPSKETIEQEKQAGE.s [1xAcetyl]	−0.107	4855.48
**Ions of y Series, (Acetylated) Thymosin *β*10 (T*β*10)**					
***m*/*z***	**Ion**	**z**	**Sequence**	**Error (Da)**	**Deconvoluted Mass**
839.5505	y14	2	p.TKETIEQEKRSEIS.	0.1142	1676.8726
592.5017	y15	3	l.PTKETIEwebQEKRSEIS.	0.1909	1773.9327
1775.0578	y15	1	l.PTKETIEQEKRSEIS.	0.1397	1790.9491
944.3809	y16	2	t.LPTKETIEQEKRSEIS.	−0.1239	1887.0094
663.8185	y17	3	n.TLPTKETIEQEKRSEIS.	0.1304	1988.0643
994.9422	y17	2	n.TLPTKETIEQEKRSEIS.	−0.0864	1988.0572
1052.2492	y18	2	k.NTLPTKETIEQEKRSEIS.	0.1992	2119.131
1244.6476	y21	2	t.QEKNTLPTKETIEQEKRSEIS.	−0.0005	2504.3272
863.8784	y22	3	e.TQEKNTLPTKETIEQEKRSEIS.	0.0947	2605.3821
1295.2735	y22	2	e.TQEKNTLPTKETIEQEKRSEIS.	0.1016	2605.3748
983.2832	y25	3	k.KTETQEKNTLPTKETIEQEKRSEIS.	0.1044	2946.5364
1130.0686	y29	3	k.AKLKKTETQEKNTLPTKETIEQEKRSEIS.	0.1194	3403.8786
1172.5977	y30	3	d.KAKLKKTETQEKNTLPTKETIEQEKRSEIS.	−0.0498	3531.9735
630.6308	y32	6	s.FDKAKLKKTETQEKNTLPTKETIEQEKRSEIS.	0.1209	3777.0594
756.3638	y32	5	s.FDKAKLKKTETQEKNTLPTKETIEQEKRSEIS.	−0.0466	3777.0525
945.1581	y32	4	s.FDKAKLKKTETQEKNTLPTKETIEQEKRSEIS.	−0.1031	3777.0452
810.8174	y35	5	e.IASFDKAKLKKTETQEKNTLPTKETIEQEKRSEIS.	0.1763	4048.2055
807.212	y35-NH_3_	5	e.IASFDKAKLKKTETQEKNTLPTKETIEQEKRSEIS.	−0.0238	4048.21
1059.3714	y37	4	m.GEIASFDKAKLKKTETQEKNTLPTKETIEQEKRSEIS.	−0.1943	4251.2934
744.6523	y39-H_2_O	6	p.DMGEIASFDKAKLKKTETQEKNTLPTKETIEQEKRSEIS.	−0.07	4480.3443
1116.7269	y39-H_2_O	4	p.DMGEIASFDKAKLKKTETQEKNTLPTKETIEQEKRSEIS.	0.1471	4479.3502
916.6534	y40	5	k.PDMGEIASFDKAKLKKTETQEKNTLPTKETIEQEKRSEIS.	0.1754	4594.421
965.1255	y42	5	a.DKPDMGEIASFDKAKLKKTETQEKNTLPTKETIEQEKRSEIS.	0.0231	4837.5425
1206.0731	y42	4	a.DKPDMGEIASFDKAKLKKTETQEKNTLPTKETIEQEKRSEIS.	−0.053	4837.5354
801.7036	y42-NH3	6	a.DKPDMGEIASFDKAKLKKTETQEKNTLPTKETIEQEKRSEIS.	0.1215	4820.5236

## Supplementary Materials

Supplementary materials can be found at https://www.mdpi.com/1422-0067/21/5/1892/s1. References [61,62] are cited in the Supplementary Materials.

## Abbreviations

AKR1A1/AKR1B1	Aldo-keto Reductases Family 1 Member(s) A1/B1
AUC-ROC	Area Under the Receiver Operating Characteristic Curve
BR-F	Breiman’s Random-Forest
KEGG	Kyoto Encyclopedia of Genes and Genomes
KODAMA	Knowledge Discovery by Accuracy Maximization Analysis
OPLS-DA	Orthogonal Partial Least Squares-Discriminant Analysis
PCA	Principal Component Analysis
PLS-DA	Partial Least Squares-Discriminant Analysis
QC-RLSC	QC-based Signal-correction Method
QCs	Quality Control Samples
UBC	Urothelial Bladder Cancer
VIP	Variable Importance in Projection
