# Supplementary material for: Malignancy Grade-Dependent Mapping of Metabolic Landscapes in Human Urothelial Bladder Cancer: Identification of Novel, Diagnostic, and Druggable Biomarkers
Source: Int J Mol Sci. 2020 Mar 10;21(5):1892. doi: 10.3390/ijms21051892 (PMC7084305; doi:10.3390/ijms21051892)
Supplement: Supplementary file 1 [file ijms-21-01892-s001.pdf]

Supplementary Materials

Malignancy Grade-Dependent Mapping of Metabolic Landscapes in Human Urothelial Bladder Cancer: Identification of Novel, Diagnostic, and Druggable Biomarkers

Aikaterini Iliou[1,†], Aristeidis Panagiotakis [1,†], Aikaterini F. Giannopoulou [2,†], Dimitra Benaki [1], Mariangela Kosmopoulou [1], Athanassios D. Velentzas [2], Ourania E. Tsitsilonis [3], Issidora S. Papassideri [2], Gerassimos E. Voutsinas [4], Eumorphia G. Konstantakou [5], Evagelos Gikas [1,‡,*], Emmanuel Mikros [1,‡,*] and Dimitrios J. Stravopodis [2,‡,*]

1 Section of Pharmaceutical Chemistry, Department of Pharmacy, School of Health Sciences, National and Kapodistrian University of Athens (NKUA), Athens 15701, Greece, katerinail@pharm.uoa.gr (A.I.); arispan@pharm.uoa.gr (A.P.); dbenaki@pharm.uoa.gr (D.B.); mrgkosm@gmail.com (M.K.)
2 Section of Cell Biology and Biophysics, Department of Biology, School of Science, National and Kapodistrian University of Athens (NKUA), Athens 15701, Greece, aigiann@biol.uoa.gr (A.F.G.); tveletz@biol.uoa.gr (A.D.V.); ipapasid@biol.uoa.gr (I.S.P.)
3 Section of Animal and Human Physiology, Department of Biology, School of Science, National and Kapodistrian University of Athens (NKUA), Athens 15701, Greece, rtsitsil@biol.uoa.gr
4 Laboratory of Molecular Carcinogenesis and Rare Disease Genetics, Institute of Biosciences and Applications, National Center for Scientific Research (NCSR) "Demokritos", Athens 15310, Greece, mvoutsin@bio.demokritos.gr
5 Harvard Medical School, Massachusetts General Hospital Cancer Center (MGHCC), Charlestown, 021004 MA, USA, ekonstantakou@mgh.harvard.edu
* Correspondence: vgikas@pharm.uoa.gr (E.G.); mikros@pharm.uoa.gr (E.M.); dstravop@biol.uoa.gr (D.J.S.)
† These authors contributed equally to this work
‡ These authors contributed equally to this work

Materials and Methods

Chemicals and reagents

DMEM culture medium was purchased from Thermo Fischer Scientific Inc.-Life Technologies-Gibco (Massachusetts, USA), while Phosphate-Buffered Saline (PBS) and all other cell-culture media and reagents were provided from Merck Millipore-Biochrom AG (Merck KgAa, Darmstadt, Germany). Deuterium oxide (D_2O) was purchased from Deutero GmbH (Kastellaun, Germany). Trimethylsilyl Propionate (TSP) was used as internal standard in NMR analysis and was purchased from Sigma-Aldrich-Merck (Darmstadt, Germany). Reserpine, yochimbine, and 4-aminophenol were used as internal standards for MS analysis and purchased from Sigma-Aldrich-Merck (Darmstadt, Germany). Chloroform, methanol, and acetonitrile were purchased from Sigma-Aldrich-Merck (Darmstadt, Germany), while formic acid was obtained from PanReac Applichem (Darmstadt, Germany). All chemicals were of analytical grade and dissolved in ultrapure water (ddH_2O). For MS analysis, all chemicals were of LC-MS (Liquid Chromatography-Mass Spectrometry) grade.

Culture Conditions

The four UBC human cell lines RT4, RT112, T24, and TCCSUP were cultured in 1x DMEM medium, being suitably supplemented with Fetal Bovine Serum (FBS), L-Glutamine, Sodium Pyruvate, Sodium Bicarbonate, Non-Essential Amino Acids, Penicillin, and Streptomycin, at 37 ^{o}C,

5% CO_2 and >95% humidity-chamber environments. The same batch of FBS [Lot: 1286 C, Catalogue number: S 0115, Volume: 500 mL, Storage: –20 °C (Exp.: 30-11-2020); Biochrom GmbH, Berlin, Germany (Merck Millipore-Biochrom AG)] (10%, v/v) was used for all the four herein examined UBC cell lines culturing and growth.

Cell Collection and Storage

For each experimental condition (cell line) examined, 10^7 UBC cells (of different malignancy grades), having been grown in high-density (high-confluence) cultures, proved capable of generating sample preparations of the high quality and quantity required, for high-resolution, high-accuracy, and high-reliability metabolomics studies. Large-scale cell-culture flasks (T75) were placed on ice and cells after being harvested through a mild scrapping process; then, they were washed twice with ice-cold 1x PBS to remove any residual medium traces. Ice-temperature conditions and solutions were used for the immediate quenching of UBC cell metabolism. Generated suspensions were centrifuged at 550 g for 5 min (+4 °C), after which supernatants were carefully aspirated and produced cell pellets were immediately stored at -80 °C untill further use. To examine growth-dependent variation of metabolite content, 10 different samples for each cell line were individually (and simultaneously) prepared.

Sample Preparation

For NMR analysis, the dried cell extracts of polar metabolites were reconstituted in Potassium Phosphate Monobasic buffer, pH 7.4, prepared in Deuterated Water [containing Trimethylsilylpropanoic Acid (TSP) as internal standard and Sodium Azide as preservative]. The use of a buffer solution ensures the stable and constant pH value of the examined samples in order to avoid signal shifts. NMR samples were dried under vacuum and washed twice with water and methanol in order to exchange the deuterated protons for MS analysis. Dried cell extracts were reconstituted with 200 μL 95 : 5 v/v water/acetonitrile, containing 10 μg/mL of internal standards. Quality Control (QC) samples were prepared by mixing aliquots (80 μL) of all analyzed samples.

Metabolite Extraction

Polar and lipid metabolites were separated using a methanol/chloroform/water (2:2:1.8) system in a two-step process, as previously described by Wu *et al*. (2008) [61].

NMR Analysis

NMR experiments were performed at the NMR Bruker AVANCE III 600 MHz Spectrometer. Proton 1D experiments were acquired using the nOesy-pre-saturation pulse sequence, with gradients (noesygppr1d, Bruker library) offering the optimum Water suppression. For each ^1H 1D spectrum, 256 scans were acquired with a spectral width of SW = 12335.526 Hz and a sampling of 64,000 points, resulting in an acquisition time of 2.7 sec. A mixing time of 10 msec was used. Sample loading, temperature stability, field homogeneity, pulse calibration, data acquisition, and processing (including Fourier transform, phase and baseline correction, and axis calibration referenced to the chemical shift of TSP at $\delta = 0.00$ ppm) were fully automated and controlled by the IconNMR v. 5.0.7 software (Bruker BioSpin GmbH, Rheinstetten, Germany). TopSpin 3.5 (Bruker BioSpin GmbH, Rheinstetten, Germany) was used for spectra visualization.

MS Analysis

For UPLC (Ultra Performance Liquid Chromatography), we used the Acquity UPLC system (Waters Corporation, Milford, USA), which was hyphenated with a highly resolved, mass accurate hybrid Ion Trap-Orbitrap Mass Spectrometer, LTQ Orbitrap Discovery XL (Thermo Fisher Scientific, Illinois, USA). ESI (Electrospray Ionization) in positive mode was applied. Chromatographic separation was performed using a C-18 column (75 μm x 50 cm; 100 A°; 2

μm-bead-packed Acclaim PepMap RSLC; Thermo Fisher Scientific, Illinois, USA) with the gradient elution previously described for metabolomics studies [62]. A QC sample was being injected every 5 consecutive samples under the same experimental conditions. Data-dependent scanning at a mass range of 100–1000 m/z was applied.

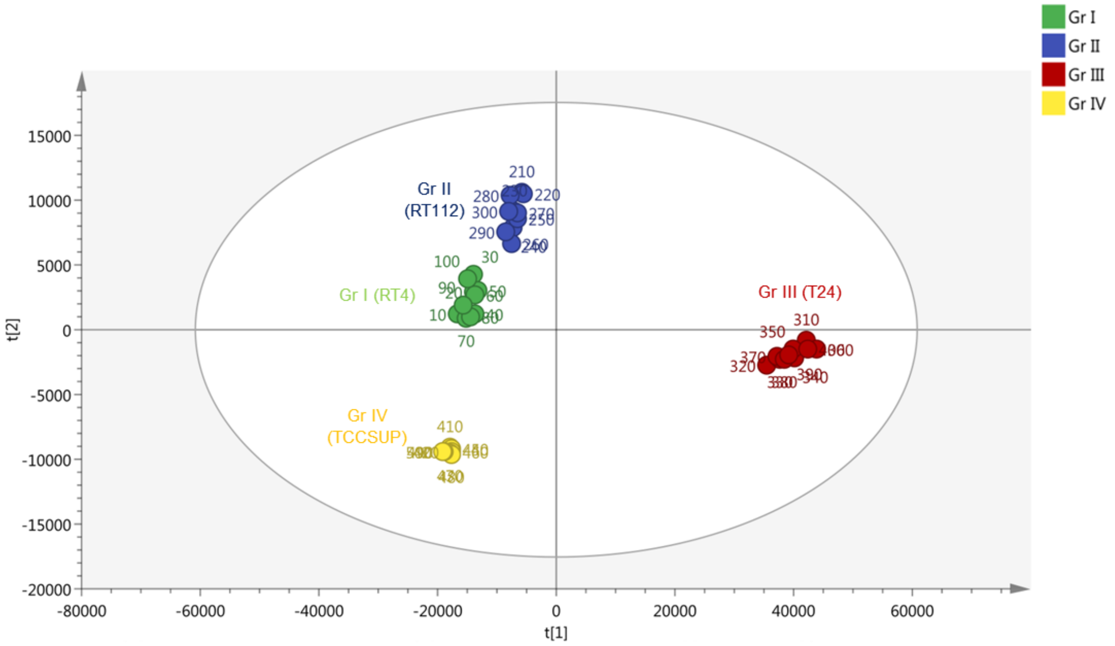

Figure S1. PC scores plot from PCA modeling of ^{1}H NMR spectra of the herein examined UBC cell lines. Green: Grade I (RT4); Blue: Grade II (RT112); Red: Grade III (T24); and Yellow: Grade IV (TCCSUP). Gr: (malignancy) grade.

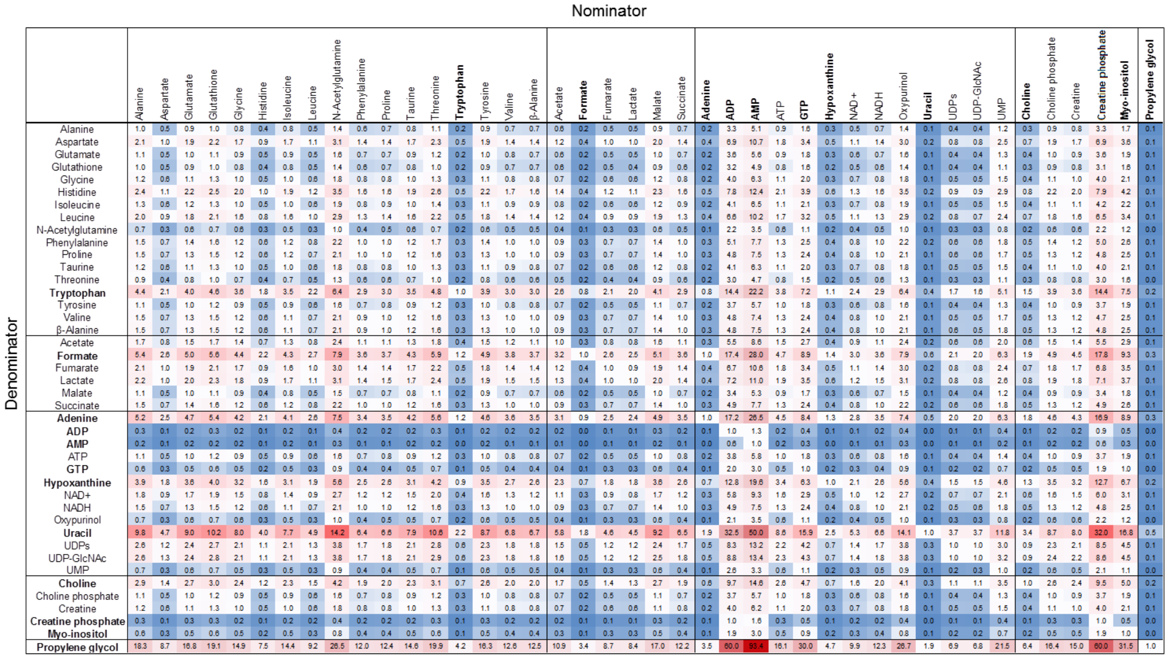

Figure S2. Heat map of the fold changes (x) of metabolic ratios of grade III (T24) versus grade I (RT4) cell group. For each pair of metabolites (nominator–denominator), the average value of the ratio in each group has been calculated. Fold changes (x) of each pair of metabolites for the grade III

(T24) group are shown, taking grade I (RT4) as the reference (control) group. Red coloring of metabolites as nominators in the ratios indicates an increase in the grade III (T24) cell group, while blue coloring indicates a decrease. The opposite principle stands for the denominators.

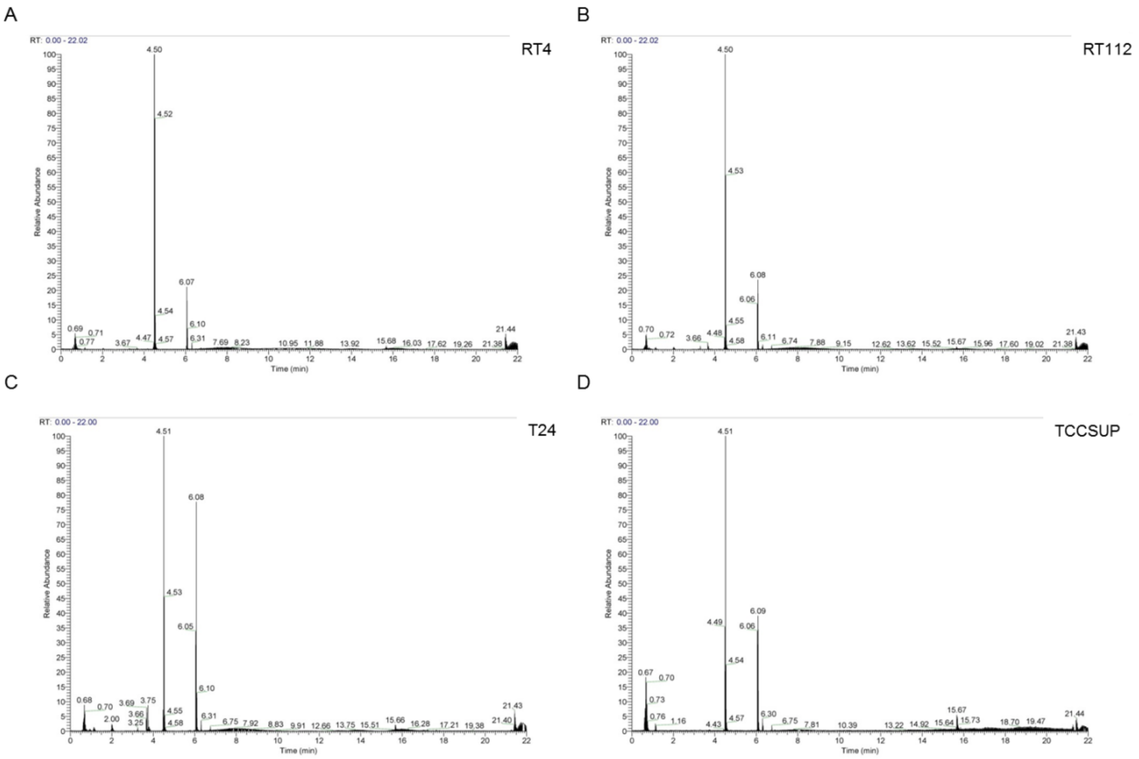

Figure S3. Representative base peak chromatograms of MS analysis, in positive mode, for the four UBC cell lines. (**A**) Grade I (RT4); (**B**) Grade II (RT112); (**C**) Grade III (T24); and (**D**) Grade IV (TCCSUP).

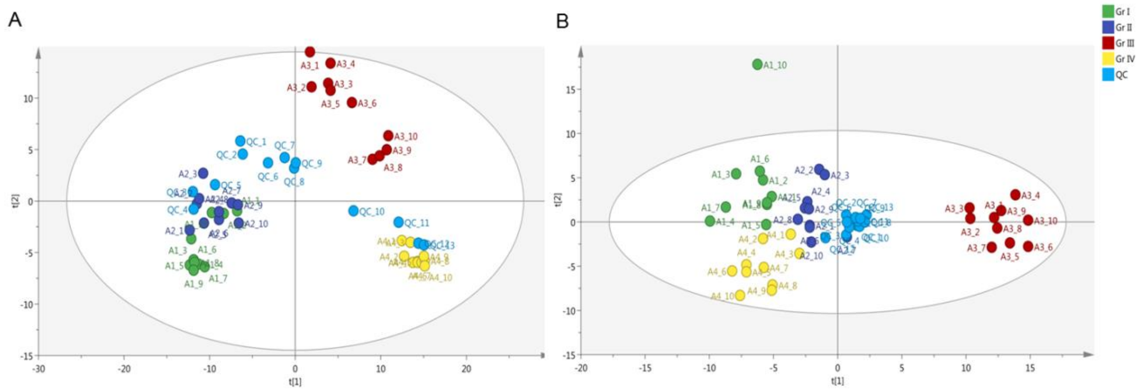

Figure S4. PC scores plot from PLS-DA modeling of MS analysis for the four UBC cell lines, before (**A**) and after (**B**) QC-RLSC correction. Green: Grade I (RT4); Blue: Grade II (RT112); Red: Grade III (T24); Yellow: Grade IV (TCCSUP); and Light Blue: QC(s). Gr: (malignancy) grade.

Table S1. Chemical shift of annotated metabolites and respective multiplicity of peaks. s: singlet; d: doublet; t: triplet; and dd: doublet of doublets (quadruplet).

Metabolites	^{1}H δ (ppm) - Multiplicity
Acetate	1.92 s
Adenine	8.25 s; 8.22 s
Adenosine Diphosphate (ADP)	8.54 s; 8.28 s; 6.15 d; 4.61 t; 4.37 m
Adenosine Monophosphate (AMP)	8.61 s; 8.28 s; 6.15 d; 4.51 dd; 4.36 m; 4.01 m
Adenosine Triphosphate (ATP)	8.54 s; 8.28 s; 6.15 d; 4.61 t; 4.40 m
Alanine	3.79 q; 1.48 d
Aspartate	3.90 q; 2.81 dd; 2.69 dd
Choline	3.20 s
Choline Phosphate	4.17 m; 3.60 m; 3.22 s
Creatine	3.93 s; 3.04 s
Creatine Phosphate	3.95 s; 3.05 s
Formate	8.46 s
Fumarate	6.52 s
Glutamate	3.76 dd; 2.36 m; 2.34 m; 2.13 m; 2.06 m
Glutathione	4.57 q; 3.78 m; 2.96 m; 2.58 m; 2.54 m; 2.19 m; 2.16 m
Glycine	3.56 s
Guanosine Triphosphate (GTP)	8.14 s; 5.92 d
Histidine	7.89 d; 7.10 s
Hypoxanthine	8.22 s; 8.20 s
Isoleucine	3.68 d; 1.27 m; 1.02 d; 0.94 t
Lactate	4.11 q; 1.33 d
Leucine	3.74 q; 1.72 m; 0.97 d; 0.96 d
Malate	4.30 dd; 2.68 dd; 2.38 dd
Myo-Inositol	4.07 t; 3.63 t; 3.54 dd; 3.29 t
N-Acetylglutamine	4.15 m; 2.07 m; 2.02 s; 1.92 m
Nicotinamide Adenine Dinucleotide (NAD+)	9.34 d; 9.15 d; 8.84 d; 8.43 s; 8.20 m; 8.18 s; 6.10 d; 6.04 d
NADH	8.48 s; 8.24 s; 6.95 m
Oxypurinol	8.21 s
Phenylalanine	7.43 t; 7.38 t; 7.34 d; 4.00 d
Proline	4.14 dd; 3.42 m; 3.35 m; 1.99 m
Propylene Glycol	3.45 dd; 1.15 d
Succinate	2.41 s
Taurine	3.43 t; 3.26 t
Threonine	4.25 m; 3.59 d; 1.33 d
Tryptophan	7.74 d; 7.55 d;7.33 s
Tyrosine	7.20 d; 6.91 d; 3.95 dd
Uracil	7.55 d; 5.81 d
Uridine Diphosphates (UDPs)	7.95 d; 5.98 d; 5.63 dd
UDP-N-Acetylglucosamine (UDP-GlcNAc)	7.96 d; 5.99 d; 5.98 d; 5.52 dd; 2.08 s
Uridine Monophosphate (UMP)	8.11 d; 5.99 d; 5.97 d
Valine	3.61 d; 2.28 m; 1.05 d; 1.00 d
β-Alanine	3.18 t; 2.56 t

Table S2. Results of different methodologies for variables' ranking for the discrimination of the four UBC cell lines. VIP scores from the PLS-DA model, loadings and Kruskal–Wallis ranking from KODAMA model and multi-ROC AUC values.

Variable ID	VIP (PLS-DA)	Loadings (KODAMA)	Kruskal-Wallis (KODAMA)	AUC	Variable ID	VIP (PLS-DA)	Loadings (KODAMA)	Kruskal-Wallis (KODAMA)	AUC
118.0643_2.01	1.095	0.120	7.226	0.922	338.174_4.5	0.657	0.093	3.427	0.767
118.0644_21.43	1.262	0.003	5.128	0.787	348.7831_0.69	1.177	0.136	9.237	0.972
118.0644_3.25	0.885	0.118	5.048	0.845	350.7812_0.69	1.079	0.125	6.777	0.910
119.0345_0.86	1.407	0.009	3.489	0.790	351.1691_20.95	0.618	0.061	3.673	0.730
120.08_2.03	0.941	0.103	7.952	0.935	352.7546_0.69	1.139	0.110	2.803	0.850
122.9237_0.69	0.780	0.096	3.963	0.735	353.1155_5.99	1.089	0.110	5.672	0.822
125.9855_21.43	1.106	0.035	1.594	0.693	355.2003_4.52	0.968	0.031	2.100	0.668
125.9856_21.85	0.570	0.011	4.288	0.618	356.2035_4.5	1.023	0.045	2.457	0.665
126.9548_21.63	0.762	0.013	2.610	0.502	359.8176_21.76	0.382	0.001	2.104	0.598
126.9713_21.41	0.477	0.017	4.890	0.662	368.4237_20.84	0.697	0.011	0.500	0.575
126.9713_21.91	0.801	0.020	1.874	0.620	368.7284_0.69	1.080	0.126	5.030	0.872
127.9661_21.6	0.951	0.013	1.371	0.563	370.7264_0.69	1.009	0.080	6.332	0.887
128.95_0.57	0.790	0.016	1.740	0.652	370.765_0.69	1.074	0.104	4.611	0.800
128.9501_21.63	0.317	0.006	2.567	0.538	380.7729_0.69	0.977	0.110	6.420	0.925
129.1015_3.71	1.012	0.136	5.060	0.980	386.7388_0.69	1.151	0.127	5.100	0.898
133.0853_5.36	1.279	0.040	2.351	0.767	388.7369_0.69	1.119	0.134	5.046	0.880
134.0593_0.92	0.483	0.003	2.392	0.567	454.7258_0.69	1.377	0.043	2.359	0.768
135.9915_21.49	0.891	0.005	0.401	0.628	464.7335_0.69	0.797	0.115	6.297	0.810
136.061_0.82	1.025	0.141	6.759	0.878	470.6995_0.69	1.018	0.120	5.222	0.782
136.061_1.15	1.356	0.012	6.530	0.792	472.6975_0.69	1.077	0.115	5.918	0.843
136.0611_3.3	2.116	0.080	7.481	0.955	484.7149_0.66	1.052	0.127	6.693	0.897
136.0749_0.98	0.795	0.126	7.407	0.882	486.713_0.66	1.112	0.124	7.849	0.902
137.9868_21.43	0.407	0.035	7.936	0.815	506.6966_0.69	0.943	0.022	1.156	0.637
137.9868_21.69	0.575	0.023	6.178	0.882	522.6707_0.69	0.991	0.103	6.313	0.848

138.9946_21.43	1.084	0.042	0.547	0.680	524.6686_0.69	0.935	0.121	7.348	0.867
138.9946_21.69	0.994	0.053	1.671	0.733	538.6866_0.69	1.325	0.058	1.677	0.693
139.0172_21.41	0.974	0.025	4.308	0.712	544.6525_0.69	1.493	0.111	9.208	0.877
139.9872_21.41	0.733	0.001	0.131	0.675	554.6604_0.69	0.973	0.106	7.518	0.885
139.9872_21.82	0.495	0.028	2.467	0.657	556.6585_0.69	0.993	0.124	5.821	0.875
140.9905_21.43	0.885	0.063	4.441	0.675	560.6266_0.69	1.020	0.123	5.032	0.872
140.9905_21.81	0.780	0.018	0.084	0.548	562.6246_0.69	1.175	0.107	4.543	0.767
141.9579_0.57	0.668	0.019	5.680	0.703	568.676_0.69	0.973	0.024	3.306	0.758
141.9579_21.79	1.228	0.032	2.006	0.725	577.4779_16.03	0.886	0.035	0.079	0.623
141.9825_21.4	1.276	0.004	0.522	0.710	590.658_0.69	0.651	0.015	0.008	0.615
141.9825_21.76	1.020	0.083	5.144	0.865	608.6301_0.69	0.417	0.058	7.666	0.787
143.0389_21.51	0.780	0.070	4.510	0.833	622.646_0.68	1.038	0.129	7.740	0.912
143.9801_21.43	0.909	0.048	2.670	0.683	637.5351_17.29	0.996	0.027	0.059	0.578
143.9802_21.85	0.753	0.045	0.674	0.653	638.6056_20.85	0.677	0.005	1.397	0.668
144.08_4.5	0.798	0.052	1.012	0.670	638.6216_0.69	0.977	0.118	6.678	0.888
144.9814_21.35	0.923	0.075	1.276	0.578	644.5876_0.69	0.969	0.119	7.820	0.893
152.0559_1.18	1.600	0.065	4.089	0.887	651.5508_17.77	0.915	0.004	0.189	0.580
152.9943_21.66	1.095	0.033	4.619	0.845	658.6033_0.68	0.898	0.119	5.720	0.882
153.0103_21.51	0.887	0.045	3.322	0.643	660.6013_0.69	1.033	0.120	5.253	0.878
153.9927_21.61	0.578	0.028	1.678	0.533	677.5664_17.82	0.816	0.047	0.041	0.558
154.9895_0.57	1.359	0.042	2.485	0.733	690.5928_0.69	0.708	0.052	1.798	0.677
154.9895_21.71	1.055	0.042	2.269	0.745	695.577_17.59	1.041	0.022	0.034	0.567
155.9926_21.67	1.142	0.006	5.576	0.767	696.5591_0.69	1.076	0.107	7.141	0.942
165.9822_21.21	0.939	0.061	2.624	0.713	698.5579_0.69	1.187	0.084	6.876	0.867
165.9822_21.6	1.051	0.029	5.471	0.752	709.5928_17.79	1.507	0.003	0.179	0.715
167.0121_21.42	0.955	0.048	2.064	0.717	719.5403_16.13	0.303	0.045	0.096	0.530
167.9813_21.57	0.430	0.006	2.184	0.687	719.5404_17.17	1.061	0.002	1.096	0.678
167.9978_21.27	0.794	0.025	0.936	0.605	721.5926_17.65	1.046	0.031	0.136	0.595
167.9978_21.82	0.558	0.004	5.558	0.628	734.5145_0.69	1.136	0.115	5.058	0.782

169.9766_21.44	1.124	0.009	5.794	0.733	739.6031_17.46	0.961	0.035	0.413	0.573
169.9766_21.7	1.096	0.021	2.601	0.735	765.6188_17.5	0.818	0.012	1.964	0.705
170.9796_21.58	0.974	0.014	1.680	0.672	796.5358_0.69	0.951	0.102	5.223	0.828
172.9556_21.49	1.117	0.028	6.470	0.738	823.4258_3.79	1.073	0.139	8.394	0.932
174.8951_0.66	0.574	0.058	1.308	0.628	823.5929_3.8	1.091	0.130	6.311	0.932
177.9569_0.79	1.267	0.072	3.676	0.792	823.7596_3.8	1.069	0.126	6.577	0.928
182.9844_21.68	0.658	0.068	4.590	0.803	823.9269_3.79	1.063	0.132	6.619	0.938
193.9293_21.57	1.134	0.011	6.790	0.738	824.0938_3.8	1.071	0.123	4.977	0.938
196.877_0.69	0.881	0.110	3.703	0.820	827.7527_3.7	0.992	0.126	4.480	0.900
203.9034_21.73	0.920	0.067	4.002	0.810	827.9193_3.71	1.025	0.131	5.044	0.952
212.1271_4.52	0.676	0.095	3.349	0.775	828.0866_3.71	1.028	0.133	5.138	0.960
212.8509_0.69	1.195	0.120	6.088	0.908	828.2532_3.71	1.019	0.135	5.135	0.982
214.849_0.69	1.090	0.126	6.216	0.855	828.4207_3.71	1.021	0.135	6.232	0.950
231.8983_21.76	1.095	0.019	4.726	0.748	828.5893_3.79	0.885	0.124	5.182	0.908
234.9559_21.6	0.872	0.041	2.780	0.568	828.7551_3.7	1.034	0.132	5.011	0.913
240.2315_6.86	0.475	0.051	4.243	0.768	955.754_15.68	1.160	0.037	0.040	0.603
250.8067_0.69	1.026	0.117	5.228	0.832	956.7576_15.68	1.193	0.042	0.081	0.678
254.9143_21.73	1.238	0.019	2.747	0.760	957.761_15.68	1.261	0.047	0.888	0.723
268.2627_7.82	0.526	0.021	1.567	0.627	971.7281_15.68	1.170	0.035	0.160	0.612
272.9248_21.72	1.089	0.023	4.714	0.715	972.7313_15.68	1.498	0.070	0.719	0.795
283.2622_20.84	0.627	0.043	1.084	0.660	973.7335_15.68	0.875	0.028	0.807	0.602
296.812_0.69	0.921	0.124	5.660	0.832	988.1106_3.8	1.066	0.126	7.465	0.917
310.8275_0.76	1.439	0.091	5.704	0.833	988.7121_3.78	1.048	0.129	5.476	0.907
311.2935_20.84	0.224	0.019	0.438	0.575	993.3025_3.7	1.035	0.136	5.218	0.973
330.7728_0.66	1.100	0.123	7.419	0.902	993.5029_3.7	1.059	0.132	5.036	0.972
332.8093_0.69	0.740	0.019	2.387	0.645	993.7033_3.7	1.062	0.132	5.187	0.995

Table S3. Top 50 MS features derived from the permutation-based variables' importance (P-value imputation) using the Random Forest algorithm (StatTarget Tool).

Ranking	Variable ID	Ranking	Variable ID
1	993.3025_3.7	26	828.7551_3.7
2	129.1015_3.71	27	638.6216_0.69
3	993.7033_3.7	28	118.0643_2.01
4	988.1106_3.8	29	988.7121_3.78
5	828.2532_3.71	30	212.8509_0.69
6	823.5929_3.8	31	350.7812_0.69
7	828.0866_3.71	32	388.7369_0.69
8	136.0611_3.3	33	193.9293_21.57
9	348.7831_0.69	34	152.9943_21.66
10	828.4207_3.71	35	554.6604_0.69
11	993.5029_3.7	36	622.646_0.68
12	827.9193_3.71	37	386.7388_0.69
13	824.0938_3.8	38	380.7729_0.69
14	828.5893_3.79	39	152.0559_1.18
15	120.08_2.03	40	141.9825_21.76
16	823.9269_3.79	41	696.5591_0.69
17	823.4258_3.79	42	470.6995_0.69
18	823.7596_3.8	43	172.9556_21.49
19	544.6525_0.69	44	524.6686_0.69
20	137.9868_21.69	45	330.7728_0.66
21	136.061_1.15	46	484.7149_0.66
22	827.7527_3.7	47	368.7284_0.69
23	644.5876_0.69	48	658.6033_0.68
24	972.7313_15.68	49	136.061_0.82
25	137.9868_21.43	50	353.1155_5.99

Table S4. Genes (38, in number) carrying alterations in both the T24 (grade III) UBC human cell lines and muscle-invasive bladder cancer (BC) patients (TCGA, Cell 2017, 413 cases, z: 1.5), with detection frequencies (%) of equal or more than 20% of the cohort cases examined. *H-RAS* is also classified as an altered critical common (shared between the T24 line and BC group) (onco)gene, albeit with reduced frequency (11%) (*data not shown*). Note the remarkably high frequency of *TP53* gene alterations (57%) in muscle-invasive BC patients.

#	Gene	%	#	Gene	%
1	***ARHGAP35***	21	20	***MCM3AP***	28
2	***ATXN2***	21	21	***MTERF4***	20
3	***CAD***	21	22	***MYCBP2***	20
4	***CDK13***	20	23	***N4BP2L2***	20
5	***CRBN***	27	24	***OBSCN***	20
6	***CSMD3***	24	25	***PCIF1***	29
7	***CUL1***	20	26	***PRDM4***	20
8	***DAXX***	24	27	***PREP***	21
9	***DHX8***	23	28	***REXO4***	24
10	***DIDO1***	30	29	***RYR1***	27
11	***EP300***	30	30	***RYR2***	23
12	***EPG5***	23	31	***SPEF2***	24
13	***EXO5***	25	32	***TAF1B***	21
14	***FAM72D***	23	33	***TM9SF2***	21
15	***FAT1***	21	34	***TP53***	57
16	***HMCN1***	27	35	***ZBED4***	20
17	***HTT***	23	36	***ZBTB5***	22
18	***KDM6A***	37	37	***ZNF585A***	21
19	***MAGEF1***	31	38	***ZNF768***	20

Table S5. Fold change (x) of grade III (T24) versus grade I (RT4) UBC cell group, with uracil serving as the metabolite of reference (control). Coloring is based on the extent of each increased (red), or decreased (blue) metabolite ratio in T24 (grade III) compared to RT4 (grade I) (reference/control) cells.

Metabolite	Fold Change (FC) (x)
Alanine	9.82
Aspartate	4.65
Glutamate	8.97
Glutathione	10.21
Glycine	7.98
Histidine	4.04
Isoleucine	7.67
Leucine	4.91
N-Acetylglutamine	14.19
Phenylalanine	6.39
Proline	6.65
Taurine	7.86
Threonine	10.64
Tryptophan	2.22
Tyrosine	8.72
Valine	6.75
β-Alanine	6.66
Acetate	5.82
Formate	1.77
Fumarate	4.64
Lactate	4.51
Malate	9.15
Succinate	6.51
Adenine	1.89
ADP	32.48
AMP	49.98
ATP	8.58
GTP	15.94
Hypoxanthine	2.52
NAD+	5.25
NADH	6.61
Oxypurinol	14.12
UDPs	3.69
UDP-GlcNAc	3.68
UMP	11.80
Choline	3.40
Choline Phosphate	8.73
Creatine	8.04
Creatine Phosphate	32.00
Myo-Inositol	16.83
Propylene Glycol	0.53
